# A quad DC source switched three-phase multilevel DC-link inverter topology

**DOI:** 10.1038/s41598-024-52605-3

**Published:** 2024-01-24

**Authors:** S. Sivamani, S. P. Mangaiyarkarasi, R. Gandhi Raj, S. Senthilkumar

**Affiliations:** 1 Department of Electrical and Electronics Engineering, E.G.S. Pillay Engineering College, Nagapattinam, 611002 Tamilnadu India; 2 Department of Electrical and Electronics Engineering, University College of Engineering, Panruti Campus, Panruti, 607106 Tamilnadu India; 3 Department of Electrical and Electronics Engineering, University College of Engineering (BIT Campus), Anna University, Tiruchirappalli, 620024 Tamilnadu India; 4 Department of Electronics and Communication Engineering, E.G.S. Pillay Engineering College, Nagapattinam, 611002 Tamilnadu India

**Keywords:** Energy science and technology, Engineering

## Abstract

The concept of an isolated DC source cascaded multilevel inverter finds good solutions for generating quality output voltage for low-medium power applications. It shapes the output voltage from three levels into a number of steps closer to a sinusoidal shape using small DC sources or batteries. Several advantages have been sighted like lower voltage stress and bearing noise, and lesser THD. However, a common issue in the MLIs is the total components required which increase with the rise in voltage levels. This paper proposes a three-phase MLI design having several isolated quad voltage source modules including an H-Bridge inverter. The design suggested claims reduced switching components for current conduction paths showing improved output quality. The operational features of the suggested MLI have been analyzed using Matlab/Simulink software, furthermore, an experimental module is constructed for demonstrating the effectiveness of the simulated results.

## Introduction

Multilevel inverters (MLI) were considered as an alternate solution for three-level inverters in terms of reduced device voltage stress, reduced electromagnetic interference (EMI), and low harmonic distortion (THD) levels. Several topologies have been suggested with isolated DC sources, clamping diodes or flying capacitors. More remarkable topologies using isolated DC sources embark free from voltage balancing issues to generate stepped voltage waveform. An MLI composed of 2 MPUCS fed with a single DC supply utilizing a high-frequency link has been suggested. The arrangement may produce up to 49 levels, where high voltage is provided using one single DC supply and lower voltage is tapped from high voltage using a high-frequency link^1^. A novel cascaded MLI is constituted using a series of several sub-modules supplied by several isolated DC sources. The topology looks broadly suitable for PV applications^2^. An MLI is formulated with a single-phase H-Bridge inverter tapped at the mid-point of each arm of the 3-level inverter to produce a stepped voltage waveform. However, the arrangement necessitates high-frequency transformers and it raises the overall costs^3^. A CHBMLI operated in trinary (1:3) voltage ratio for grid-connected loads is developed using a single input multi-output single-ended primary inductor (SEPIC) converter with 2-stage H-bridge inverters. The inverter system is potentially appropriate for grid-connected applications^4^. A combination of T-type as well as H-bridge inverters for producing nine-level output voltage has been suggested^5^. The topology uses capacitors and two DC sources in their modules together with DC-offset have been added to the sine reference to balance the capacitor voltages^5^. An H-6 inverter with DC sources on either side of it to produce a stepped voltage waveform is presented^6,7^. The arrangement shall be cascaded for producing additional voltage levels and it has added capacitors with a rise in voltage level count. A CHBMLI using one DC source is presented for reducing the DC sources required for creating multilevel voltages. The main H-Bridge was powered by DC supply and the left behind H-bridges were substituted using DC-link capacitors. The capacitor voltage is naturally balanced by exploring space vector redundancies and open-loop control^8^. A three-phase MLI utilizing capacitor DC-link in series with a switch is developed to generate stepped waveforms and the capacitor count increases with the number of voltage levels^9^. A three-phase MLI utilizing half-bridge cells has been formulated for offering reduced switching components, wherein three numbers of single phase are connected in star connection to configure a three-phase MLI. The number of switches in the current conduction route increases with voltage levels^10^. A series of H-Bridge inverters commonly share a single DC source and the outputs are coupled through isolation transformers to generate multilevel voltage. However, the output transformers increase the overall cost^11^.

A hybrid MLI structure for three three-phase supply is realized using three-phase 2-level and two numbers of single-phase H-Bridge inverters^12–14^. The topology uses a single DC source and tiny transformers^3^. A switched capacitor MLI is developed from half-bridge cells with a single DC source. The suggested topology overcomes the necessity of enormous switches as well as DC supplies for higher voltage levels. The suggested topology avoids high inrush current in capacitors^15^. This new topology uses 8 switching devices and 4-DC sources for generating 15-level in the output terminals. This arrangement shall be used in renewable energy mechanisms and several cells are added to boost the voltage levels^16^. A connected single-phase H-Bridge and Half bridge cells form MLI is suggested. The half-bridge cells serve as a level-doubling network in its mode of operation in the MLI structure. The suggested topology has the advantage of reactive power handling and fast fault-blocking capability^17^. A novel arrangement of bidirectional multilevel converter for electric vehicles is presented using DC-link capacitor voltage balances. The presented converter uses an additional 2 switches for capacitor voltage balance in a T-type configuration compared with conventional topology^18^. An enhanced configuration for asymmetric MLI using capacitors as DC-links to synthesize staircase waveform is developed^19^. The circuit comprises 2 chargeable capacitors and 14 semiconductor switches. The structure claims its advantage in that it does not require any charging circuit for charging the capacitors. A novel flexible architecture has been suggested^20^ that performs multilevel operations using the static synchronous compensator (STATCOM) using traditional three-phase voltage source inverters. The inverters are coupled as a cascaded design having three different DC lines and one open-end winding structure that connects to a transformer. To get a stepped voltage waveform, an H-bridge inverter is linked to a transistor-clamped circuitry having DC-link capacitors on both sides, as presented in^21^. The structure requires several bidirectional switches to allow bidirectional current flow.

A modified 3-phase DC-link multilevel design is suggested for the usage of reduced DC sources. It is formed with several half-bridge modules, H-bridge inverters, and output transformers to procure stepped voltage^22,23^. To ensure a freewheeling current route under dead time enabling an easy transition among various voltages and avoiding fluctuations in voltage, two balanced compact-module designs for cascaded MLI are devised^24^. A new MLI for grid interface has been developed using an H-Bridge inverter with less number of switching devices. The inverter requires a lesser switch count compared with traditional inverters and a simple control technique is embedded with a control method to balance the capacitor voltages and the design may not necessitate automatic controlling to balance the capacitor voltages^25^. A new topology is suggested for cascaded transformer-based MLI to offer reduced switching devices and DC sources. The topology looks simple in structure and control^26^. A three-phase topology is constituted using a traditional three-phase inverter and half-bridge cells to make a stepped voltage waveform. Several half-bridge cells are coupled to raise the voltage levels^27^. An inverter structure for achieving higher voltage levels is obtained by stacking two five-level inverters. Capacitors are needed by the inverter to produce voltage steps, therefore those voltages are balanced using a suitable regulation technique^28,29^. An improved H-bridge using an individual DC supply on one side and split capacitors with another DC source on the other side is suggested to offer lesser power components. The topology is connected to a three-phase inverter to produce three-phase voltages which makes its construction simpler in three-phase applications^30^. Each complete bridge inverter coupled in serial to a level doubling network makes up a topological architectural element. It tries to expand the diversity of levels at the output voltage waveform by connecting these construction parts^31–33^.

This work presents a novel topological structure for three-phase applications with different modes of operation for inductive load. Following with, the merits of the suggested design are then examined with comparison with respect to total power components and power loss calculation with recent topologies is presented. Subsequently, a demonstration setup is constructed to show the effectiveness of the simulated findings, as well as the efficiency of the designed structure is evaluated using simulations by MATLAB/SIMULINK.

## Proposed topology

**Figure 1:**
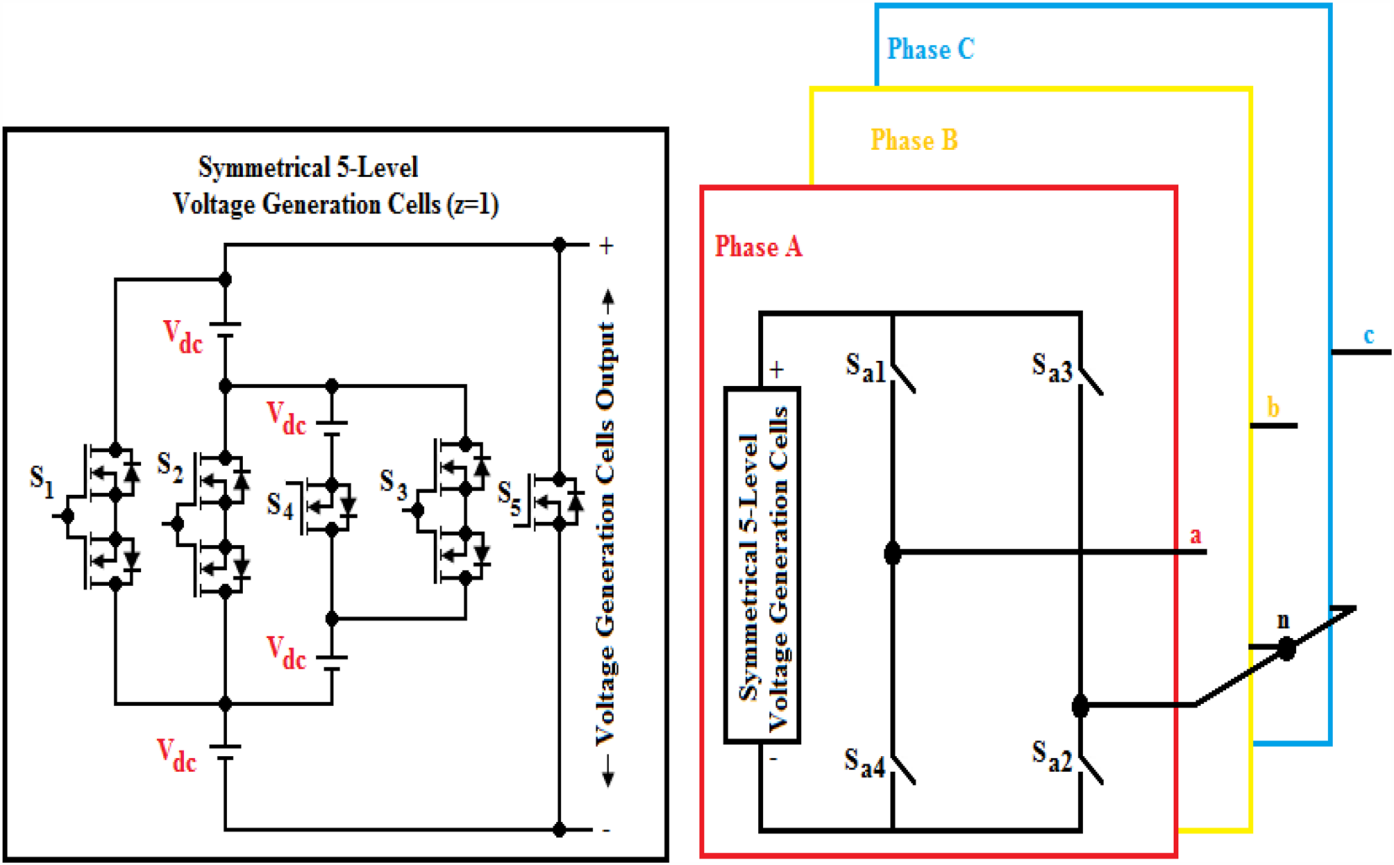
Proposed topology.

**Figure 2:**
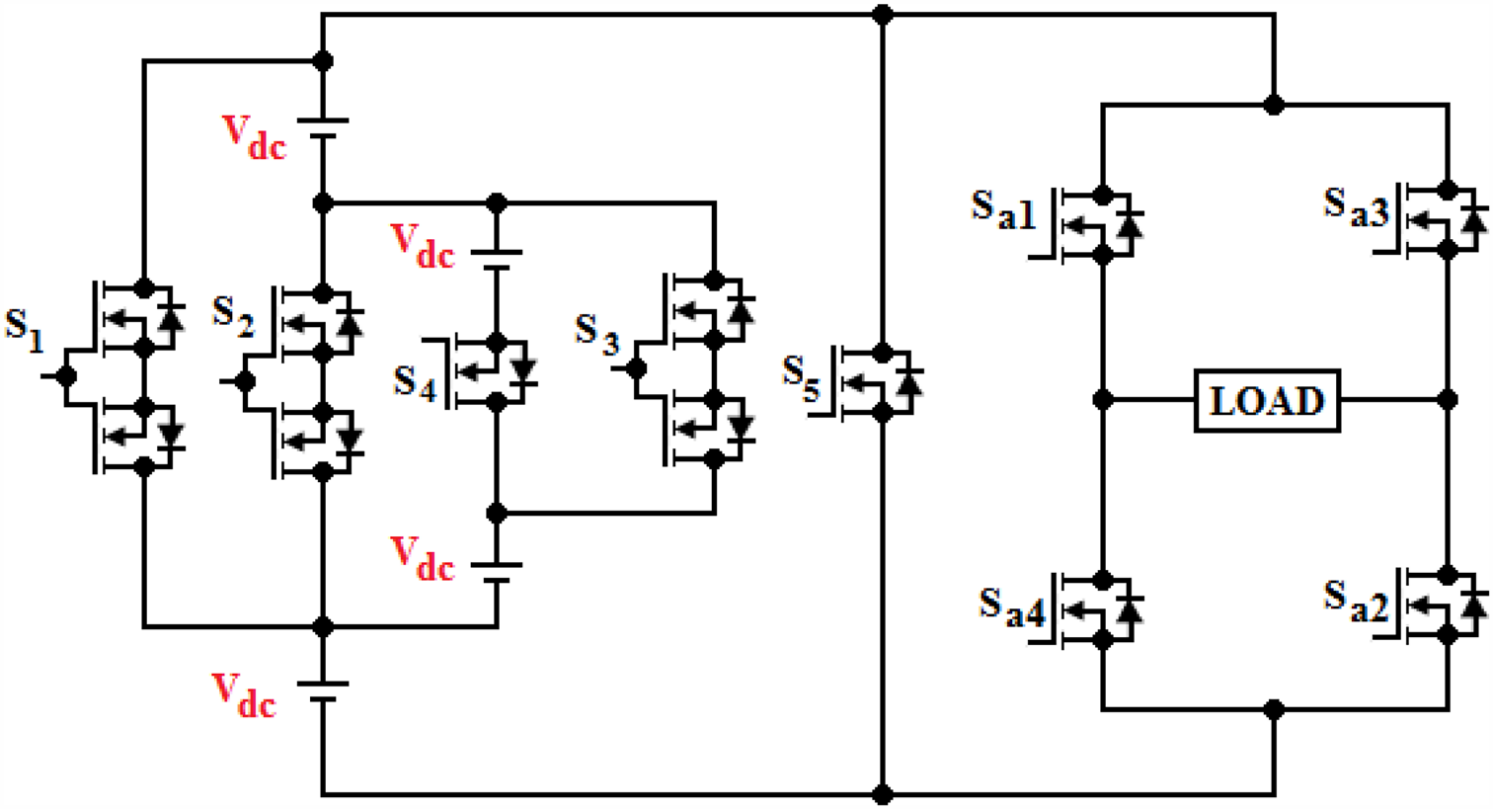
Single-phase topology.

**Figure 3:**
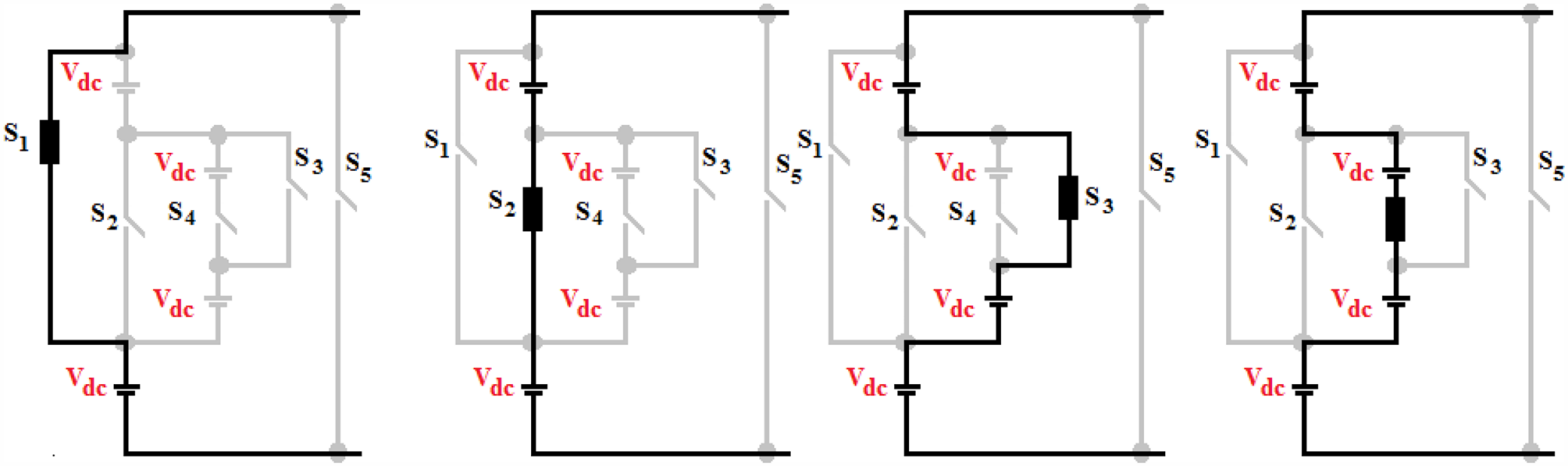
Operating modes for all voltage levels.

Figure 1 proposes a new topology for MLI to offer reduced power components compared to traditional inverters. The CHBMLI is mostly employed in high or medium (> 3 kV) settings because of their ability in generating stepped voltage using smaller DC voltage generators as well as only block voltage that is restricted to the supplied DC voltages. However, when the potential levels increase with a rise in H-Bridge inverters that leads to higher requirements of the number of devices for switching as well as DC suppliers. Hence, the paper targets to archive a new MLI configuration to prevail over the above-mentioned drawbacks. The proposed topology is composed of four voltage sources and four switching devices (MOSFET or IGBT) as voltage generation modules to play a role in generating voltage steps, and an H-Bridge inverter serves to function as a polarity reversal. Several modules are cascaded at the input side to offer higher voltage levels. Each module is capable of generating five voltage ranges (+ 4V_dc_, + 3V_dc_, + 2V_dc_, + V_dc_, 0) at the source terminal of the H-Bridge inverter, and at the load terminals with polarity reversal to acquire 9- level (± 4V_dc_, ± 3V_dc_, ± 2V_dc_, ± V_dc_, 0)), respectively. The functionality of the developed three-phase topology is understood easily by considering a single-phase design. The power circuit layout for the single-phase design and the operational modes to obtain dissimilar levels of voltage have been portrayed in Figs. 2 and 3, correspondingly.

It is observed in Fig. 3 that only one switch is required to conduct all voltage levels in the voltage generation part and two switches in the polarity reversal part. It is in contrast with traditional MLI (CHBMLI), the suggested design necessitates 3 switching conducting elements alone in the current paths, while in CHBMLI, eight switches are needed in the current paths. If the suggested design is operated with PWM, the voltage generation part is required to be PWM modulated and the H-Bridge inverter is interchanged at fundamental switching. Consequently, the suggested design claims to have less power loss than CHBMLI. The relation between the variety of voltage ranges, switches and DC sources is expressed by [(8 × z) + 1], [(4 × z) + 4] and [4 × z], where, ‘z’ is the voltage generation module respectively. Tables 1 and 2 tabulate the mathematical relations to attain various design parameters for proposed and CHB MLI topologies.

**Table 1:** Comparative analysis of the suggested and traditional single-phase designs having “p” cells.

Multilevel inverter structure	CHB topology	Topology R^39^	Proposed
Voltage levels	(2 × p) + 1	(8 × p) + 1	(8 × p) + 1
Maximum output voltage	(p) × V_dc_	(4 × p) × V_dc_	(4 × p) × V_dc_
Main switches	(4 × p)	(12 × p)	(8 × p) + 4
Gate drivers	(4 × p)	(9 × p)	(5 × p) + 4
Maximum device count in the conduction path	(2 × p)	(3 × p)	(p) + 2
SDCs	(p)	(4 × p)	(4 × p)
Overall standing voltage on switches	(4 × p) × V_dc_	(18 × p) × V_dc_	(6 × p) × V_dc_
Voltage level generation section			
Polarity reversal section			(16 × p) × V_dc_

**Table 2:** Comparative analysis of the suggested and traditional three-phase designs using “p” cells.

Multilevel inverter structure	CHB Topology	Topology R^39^	Proposed
Voltage levels	(2 × p) + 1	(8 × p) + 1	(8 × p) + 1
Main switches	3 × ((4 × p))	3 × (12 × p)	3 × ((8 × p) + 4)
Gate drivers	3 × ((4 × p))	3 × (9 × p)	3 × ((5 × p) + 4)
SDCs	3 × (p)	3 × (4 × p)	3 × (4 × p)

The driver consists of five Numbers of MOSFET/IGBT with gate driver IC’S. The gating signals are given as an input from an external control module.

- One Number of High speed opto—isolator provided for PWM isolation
- One Number of MOSFET–IRF 540-with suitable snubber circuit & Heat sink provided for
- Power circuit (optional IGBT also used if required)
- Rating of device is 400 V DC@5A
- Isolated + 12vdc@500 mA provided for control IC’s
- 230 V AC input, one number of power ON/OFF switch with indication.

## Specifications of driver circuit:

- Power circuit input: 230vac (externally)
- Power Circuit Output: 100 V Vac, 2A, Suitable for laboratory level RL load
- PWM input: 5 Numbers of PWM—5VDC level
- Protection: 2A.

**Figure 4:**
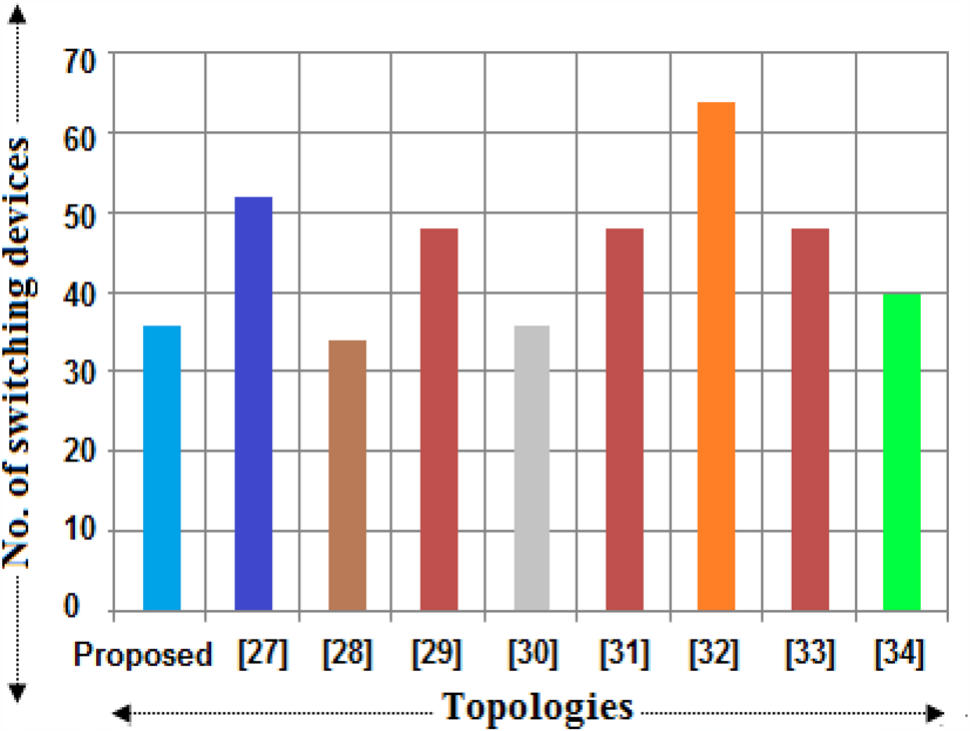
Comparison chart for switching devices requirement of proposed topology against recent MLIs^34–41^ for 33-level inverter.

**Figure 5:**
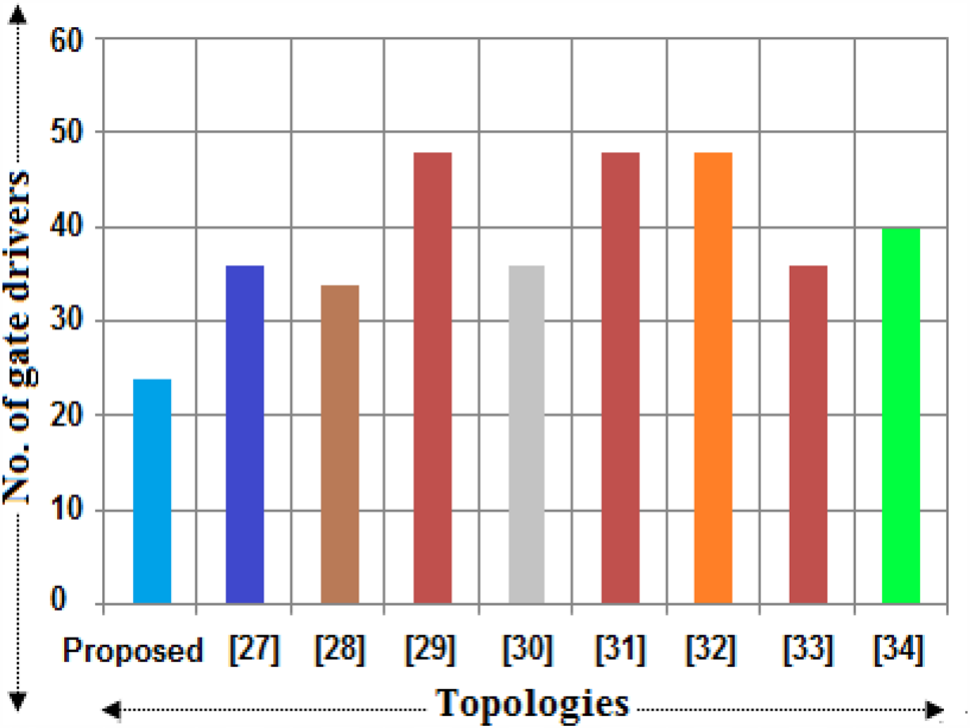
Comparison chart for gate drivers requirement of proposed topology against recent MLIs for 33-level inverter.

**Figure 6:**
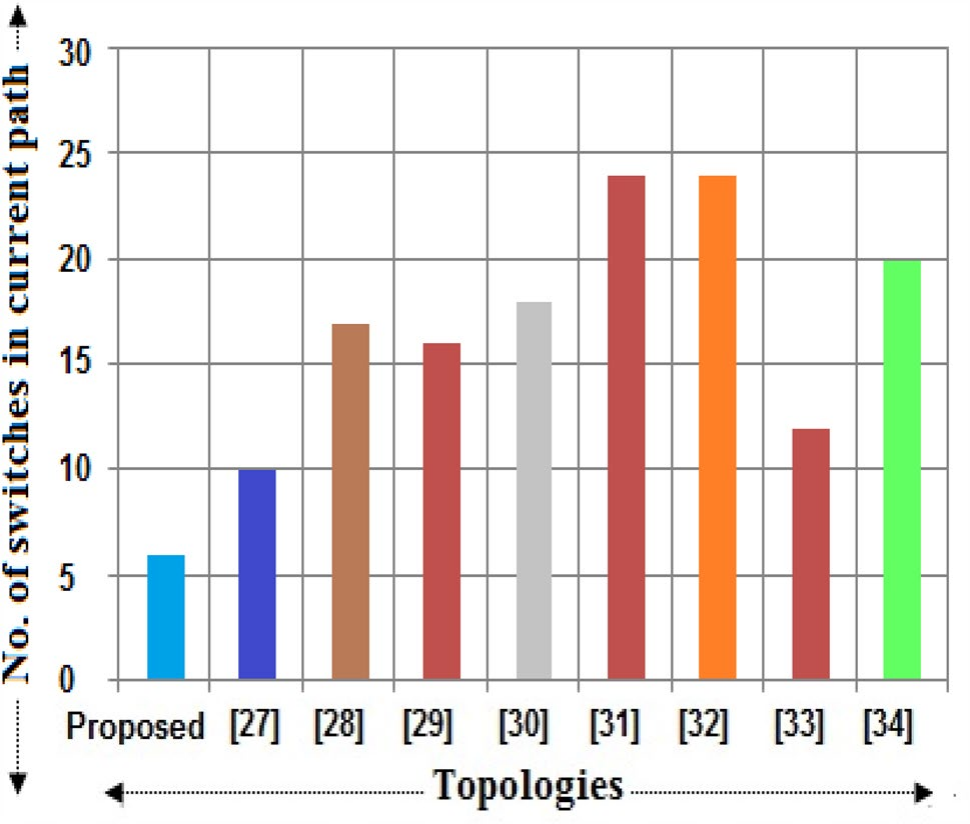
Bar chart for maximum current conducting devices of proposed topology against recent MLIs for 33-level inverter.

**Figure 7:**
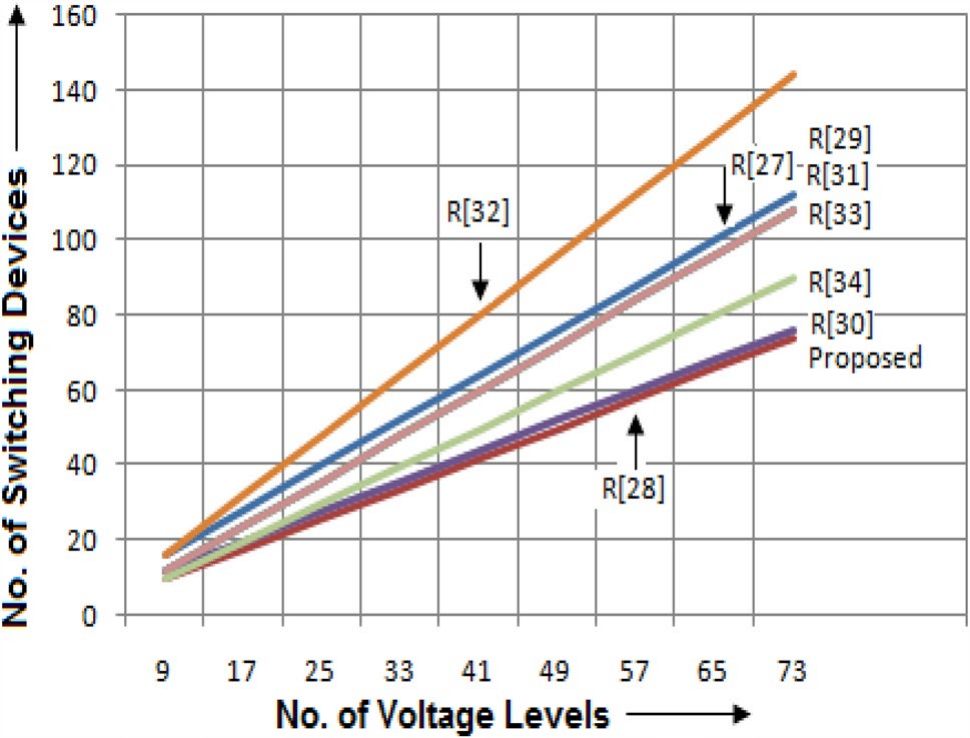
Plot between suggested and existing MLIs in terms of switching devices.

**Figure 8:**
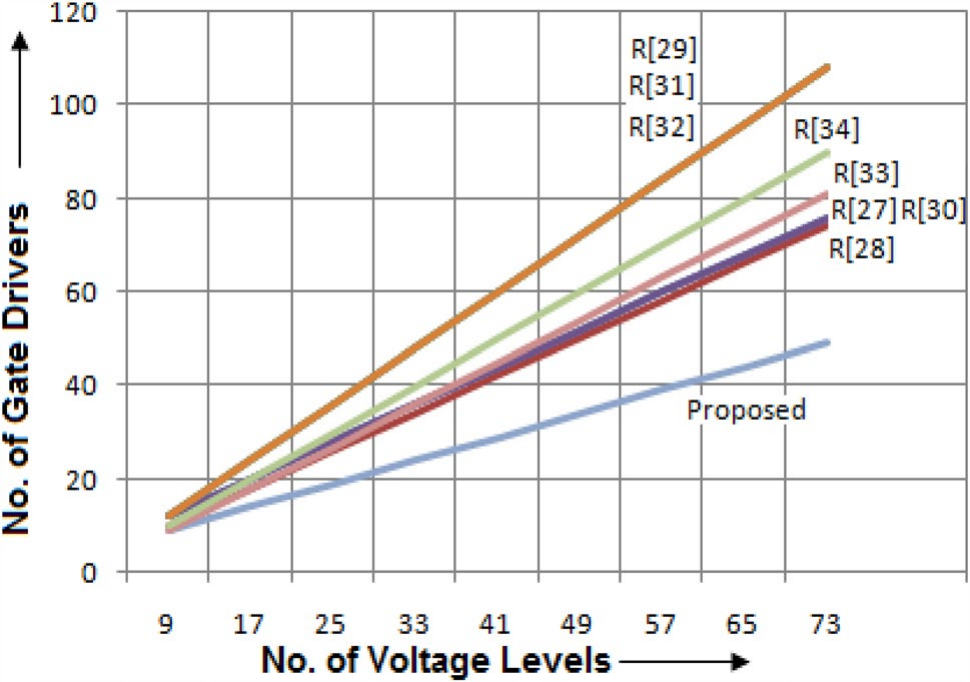
Plot between proposed and recent MLIs in terms of gate drivers.

It is required to differentiate the state-of-the-art MLIs according to several characteristics for clarifying the scenarios of the suggested single and three-phase topologies for applications in real-time. In this way, a detailed study has been portrayed in terms of recently developed single-phase units, since the suggested design needs minimum switching devices compared to recent designs. The switching devices, gate drivers, along with the overall current conducting elements for the 33-level inverter are displayed in Figs. 4, 5, and 6. It is observed from the charts that the suggested design necessitates fewer power elements concerning high voltage levels. Figures 7 and 8 signify the assessment of existing and suggested MLI through switching devices and gate driver units. The suggested MLI necessitates fewer switching elements over the current conduction path against recent MLIs as apparent in Fig. 3. It is proven that the proposed structure has the least device count in the current conduction path than other recent MLIs.

Power loss is another crucial factor that has been taken into account while analyzing the created topology’s functionality. The suggested architecture can deliver greater efficiency indices if it additionally delivers reduced power loss. Switching loss along with conduction loss account for the majority of the power loss. Every power device (P_sw_) has switching loss during turn-ON and turn-OFF. To compute switching loss, find the appropriate turn-ON and turn-off moments across a single benchmark interval in the manner of:

1 $${\text{E}}_{{\text{off,k}}} = \frac{{1}}{{6}}{\text{V}}_{{\text{sw,k}}} {\text{It}}_{{{\text{on}}}}$$

2 $${\text{E}}_{{\text{on,k}}} = \frac{{1}}{{6}}{\text{V}}_{{\text{sw,k}}} {\text{I}}^{\prime } {\text{t}}_{{{\text{on}}}}$$

3 $${\text{P}}_{sw} = f\left[ {\sum\limits_{k = 1}^{{N_{switch} }} {\left( {\sum\limits_{i = 1}^{{N_{on,k} }} {E_{on,ki} } + \sum\limits_{i = 1}^{{N_{off,k} }} {E_{off,ki} } } \right)} } \right]$$

The formula to calculate conduction losses for distinct switching devices shall be expressed as,

4 $${\text{P}}_{C} = \left( {\left[ {V_{T} + R_{T} i^{\beta } (t)} \right] + \left[ {V_{D} + R_{D} i(t)} \right]} \right)i(t)$$

**Figure 9:**
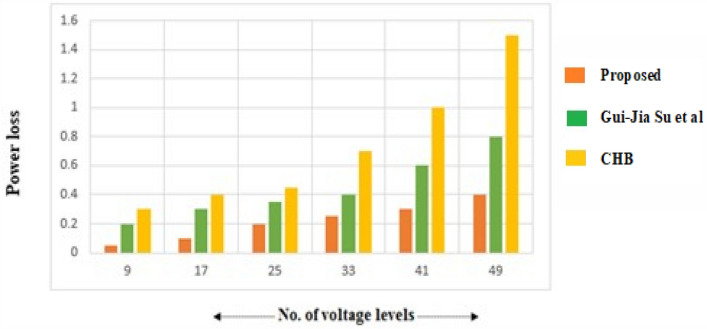
Comparison of device power loss for various voltage levels in recent topologies vs proposed topology.

The Fig. 9 illustrates the plot in terms of normalized power loss between the proposed and traditional topologies by Gui-Jia et al.^42^. and Cascaded H-Bridge MLI. It is observed from Fig. 9 that the developed MLI attains lesser power loss than the topologies by Gui-Jia et al.^42^. and CHB. For understanding the total devices in current conduction path to attain 9-level, the developed topology has 4 switching devices, while, the topologies by Gui-Jia et al.^42^. and CHB have 6 and 8 switching devices. It is inferred from the Fig. 9, the developed topology claims minimum power loss in comparison with traditional topologies with increase in voltage levels.

## Simulation results

**Figure 10:**
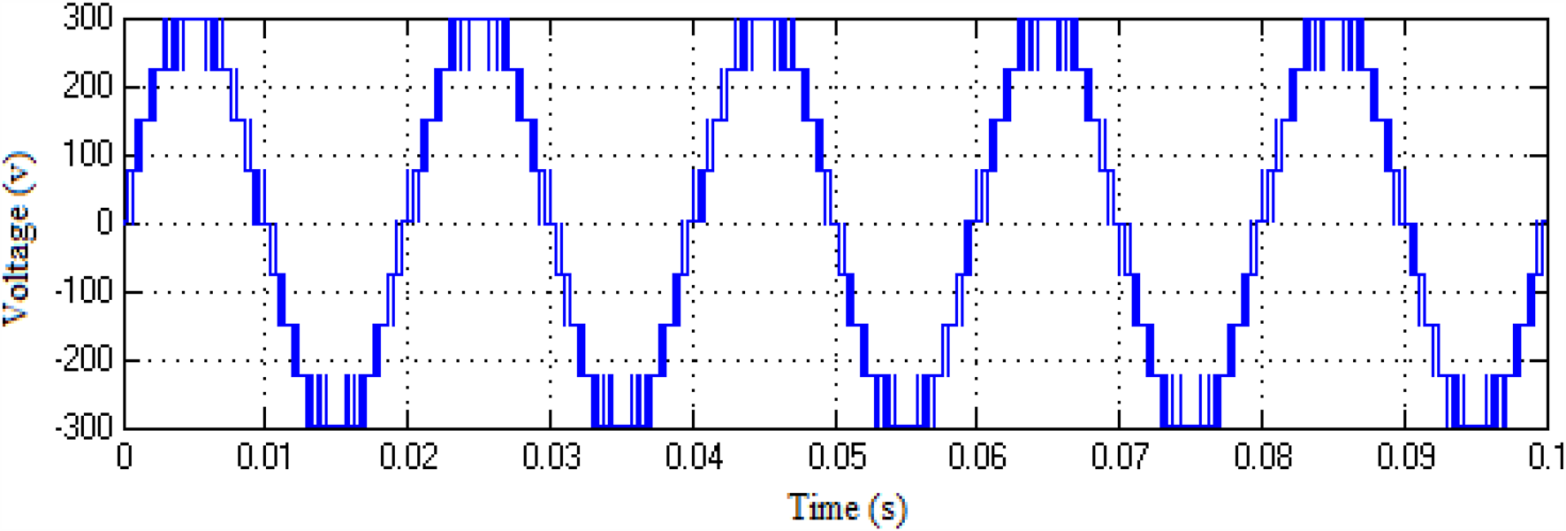
9-level inverter—output voltage.

**Figure 11:**
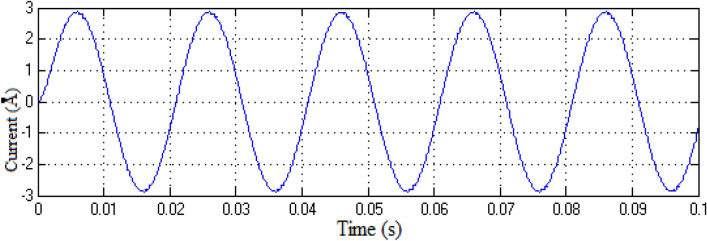
9-level inverter—inductive load current.

**Figure 12:**
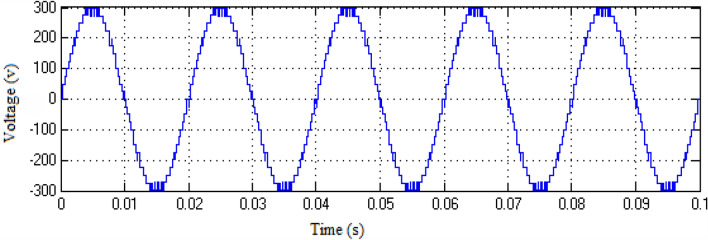
25-level inverter—output voltage.

**Figure 13:**
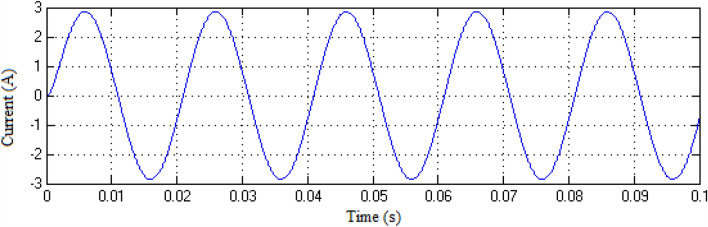
25-level inverter—inductive load current.

**Figure 14:**
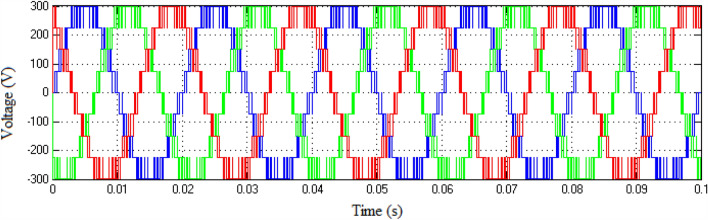
9-level inverter—phase voltage.

**Figure 15:**
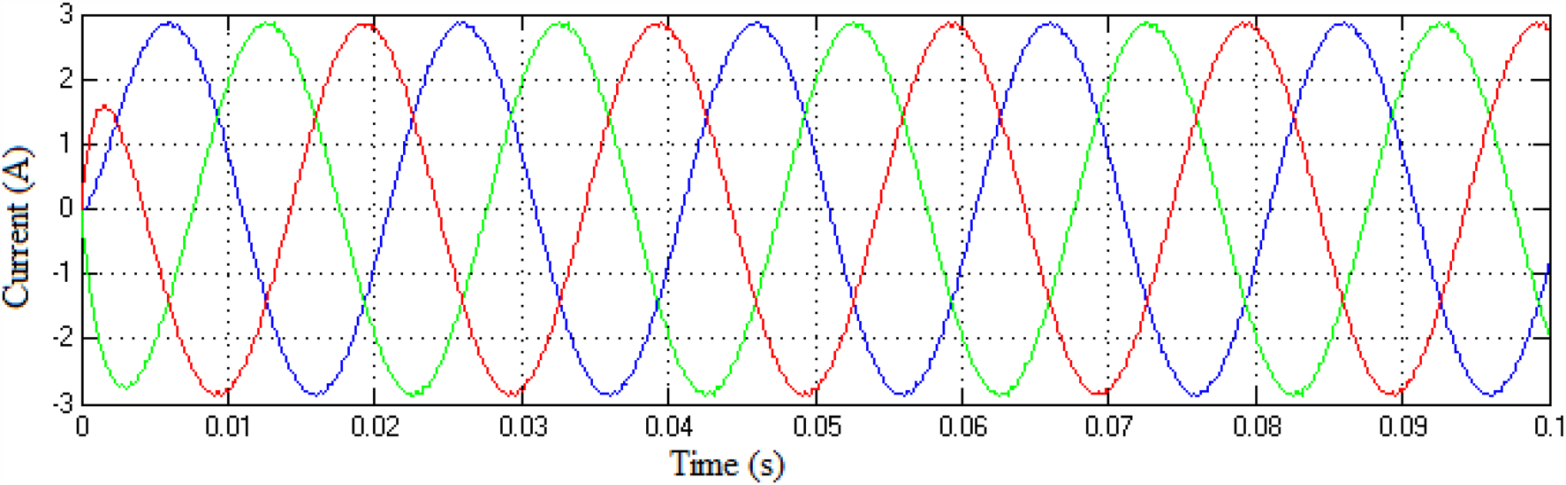
Three-phase 9-level inverter—inductive load current.

The Matlab/Simulink platform is employed to implement the functionalities provided by the suggested MLI utilizing an RL-load of 100 Ω, and 100 mH, with input DC voltage sources of 75 V respectively. The design suggested is set up to function in single-phase structures with 9 and 25-level inverters. The switching patterns used to acquire the desired output voltage are obtained through the MCPWM scheme having a carrier frequency of 2 kHz with an output frequency of 50 Hz. The output voltage along with inductive current waveforms for 9- and 25-level inverters, correspondingly, are shown in Figs. 10, 11, 12, and 13. Similarly Figs. 14 and 15 represent phase voltage and inductive load current outputs for the three-phase 9-level inverter.

## Experimental results

**Figure 16:**
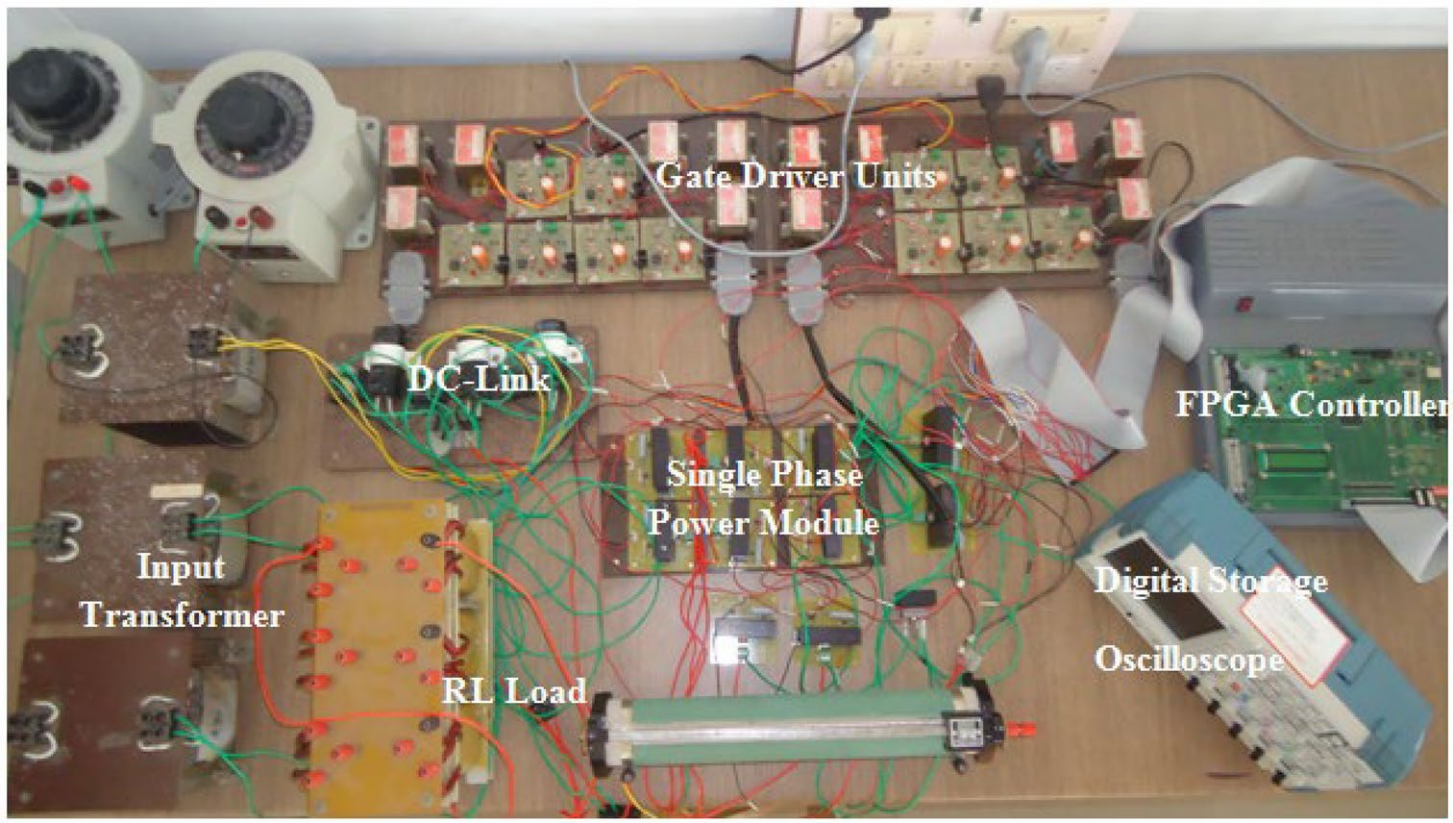
Experimental prototype.

**Figure 17:**
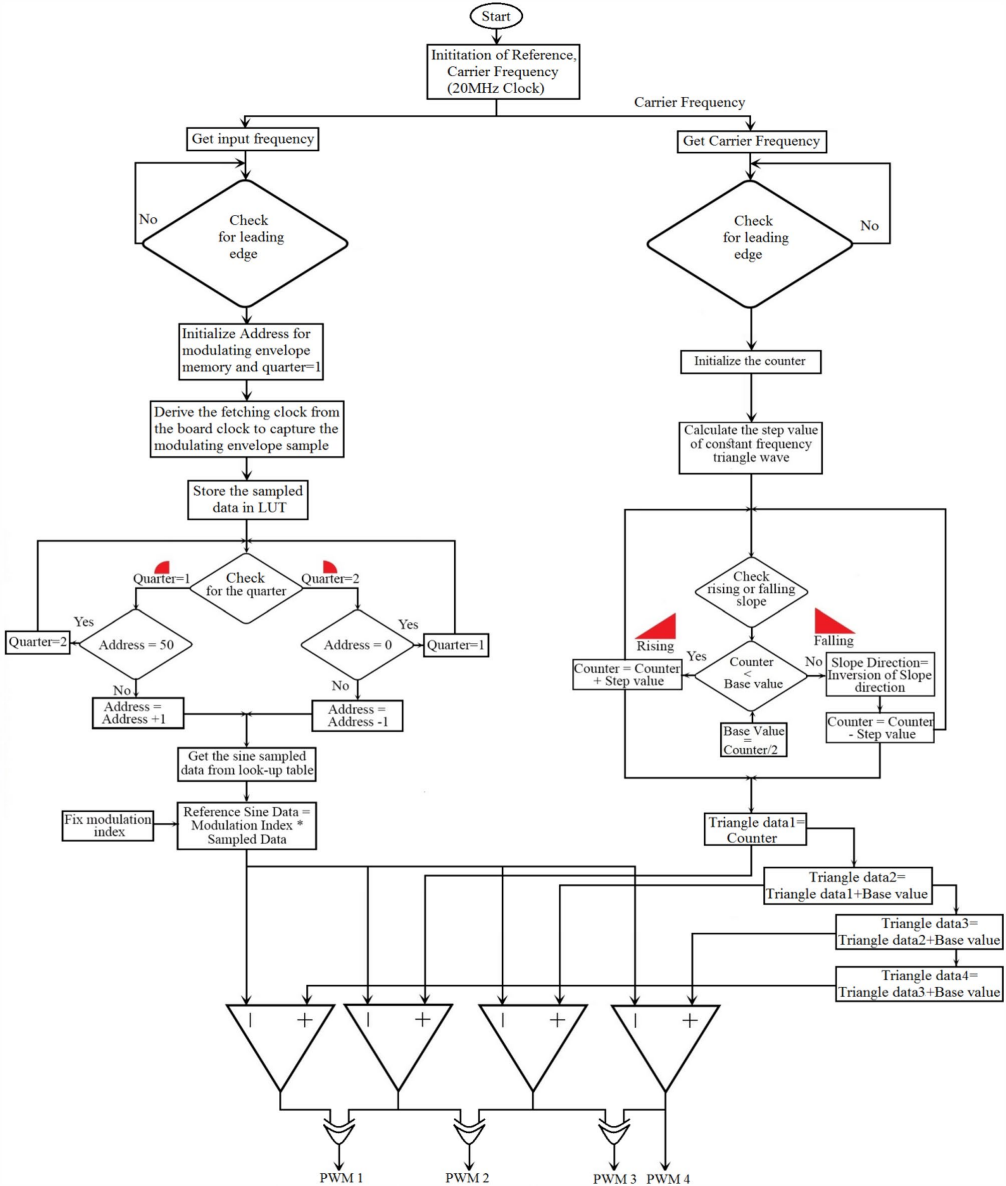
Flowchart for FPGA implementation.

**Figure 18:**
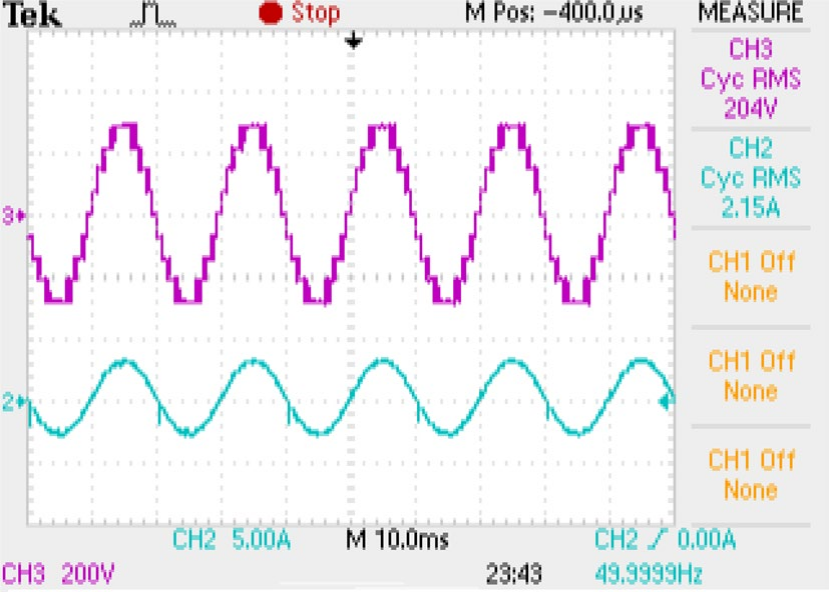
Waveforms of the output voltage of 9-level inverters along with inductive load current.

**Figure 19:**
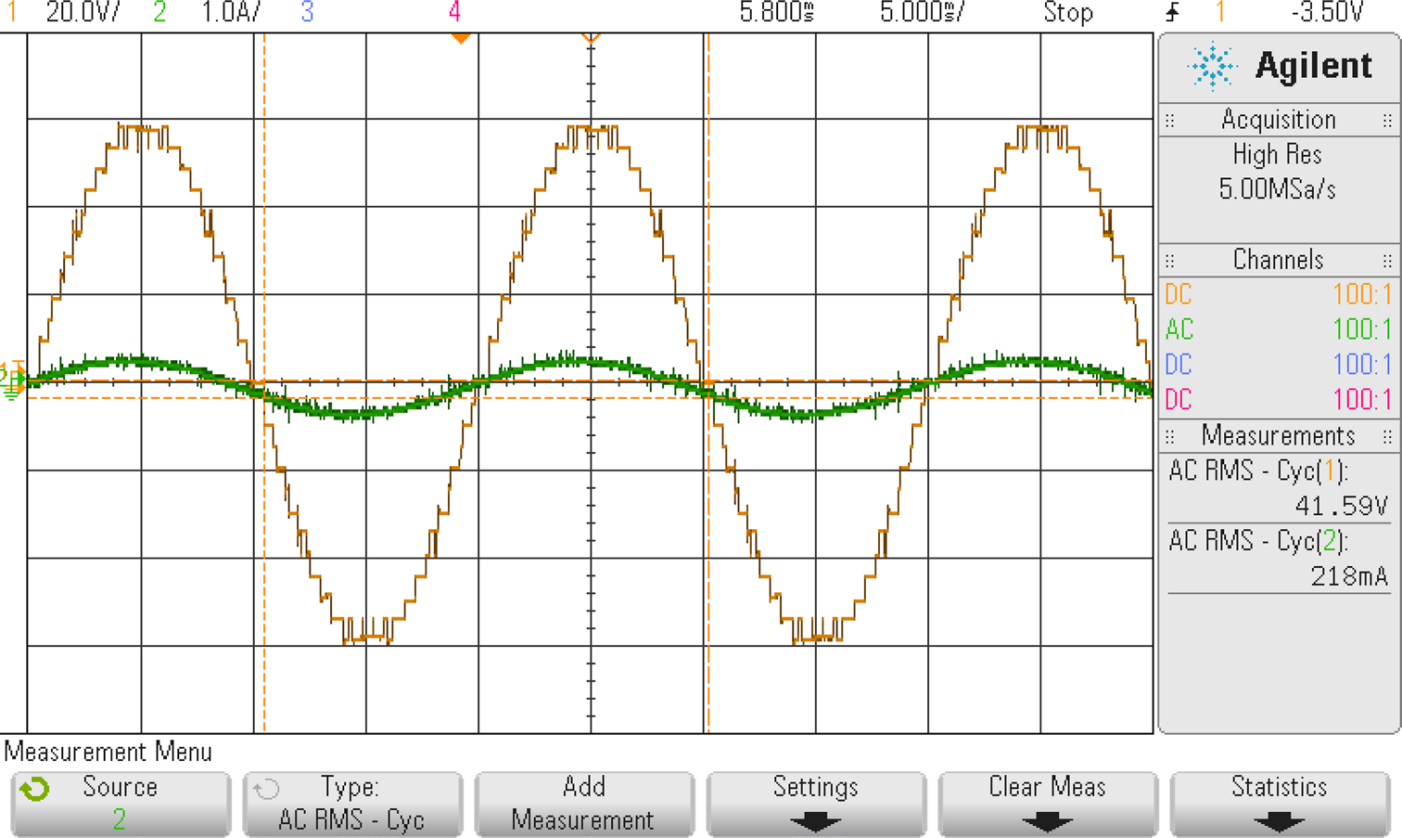
Waveforms of output voltage along with inductive load current for a 25-level inverter.

With an RL load of 150 Ω and 106 mH, the test arrangement illustrated in Fig. 16 is used for obtaining an output voltage of 300 V (peak) for 9-level and 60 V (peak) for 25-level inverters, therefore verifying the practical practicality of the suggested MLI. The test rig uses an Insulated Gate Bipolar Transistor (FSBB20CH60 IGBT) with required gate drive circuits to form the power module. The MLI avails a Multicarrier PWM strategy having a carrier frequency of 2 kHz for obtaining the required PWM pulses for generating PWM modulated stepped voltage. The FPGA flowchart for PWM generation is represented in Fig. 17. The method uses sampled sine reference and triangle carrier as Look-Up Table (LuTs), and it is regularly fetched at required cycles to generate required PWM pulses. The developed algorithm for the FPGA controller generates pulses similar to the simulated pulses in applications that operate in real time. Figures 18 and 19 show the acquired experimental results from the developed prototype and PWM pulses from the FPGA controller of the suggested design. The concept of MLI for applications that run in real-time is suggested by the test responses, which are reflected in simulation findings.

The experimental response translates the simulated results and suggests the proposed MLI for real time applications. Table 3 shows the Comparison of 9-level MLI under symmetrical operation.

**Table 3:** Comparison of 9-level MLI under symmetrical operation.

Topologies	No. of DC sources	No. of switches	No. of gate drivers	No. of max switches in current conduction path
Topology 1^43^	4	12	10	4
Topology 2^44^	4	10	9	4
Topology 3^45^	4	12	10	4
Proposed topology	4	12	10	4

The comparison is made for 9-level under symmetrical operation. The proposed and the recent topologies require same number of power components in single phase topology. However, the proposed topology requires 1/3 of total DC sources required to produce the same number of voltage levels as compared with recent topologies in case of three phase operation.

**Figure 20:**
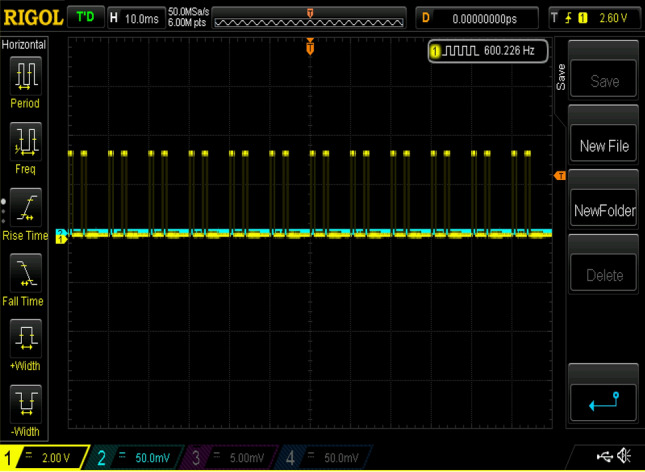
Experimental gating pulses for switch S1.

**Figure 21:**
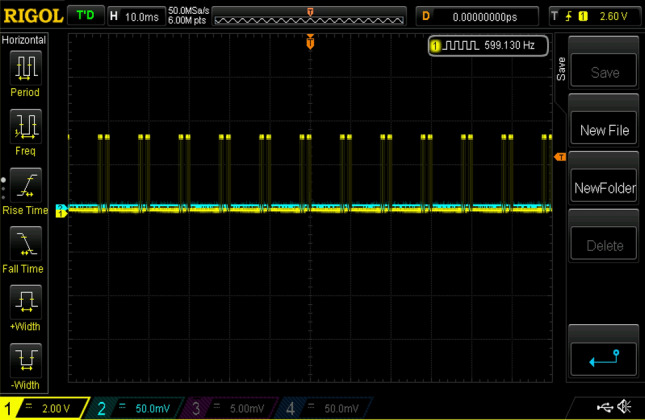
Experimental gating pulses for switch S2.

**Figure 22:**
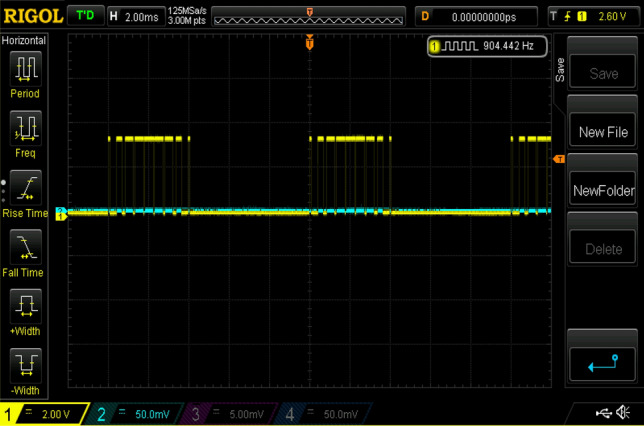
Experimental gating pulses for switch S3.

**Figure 23:**
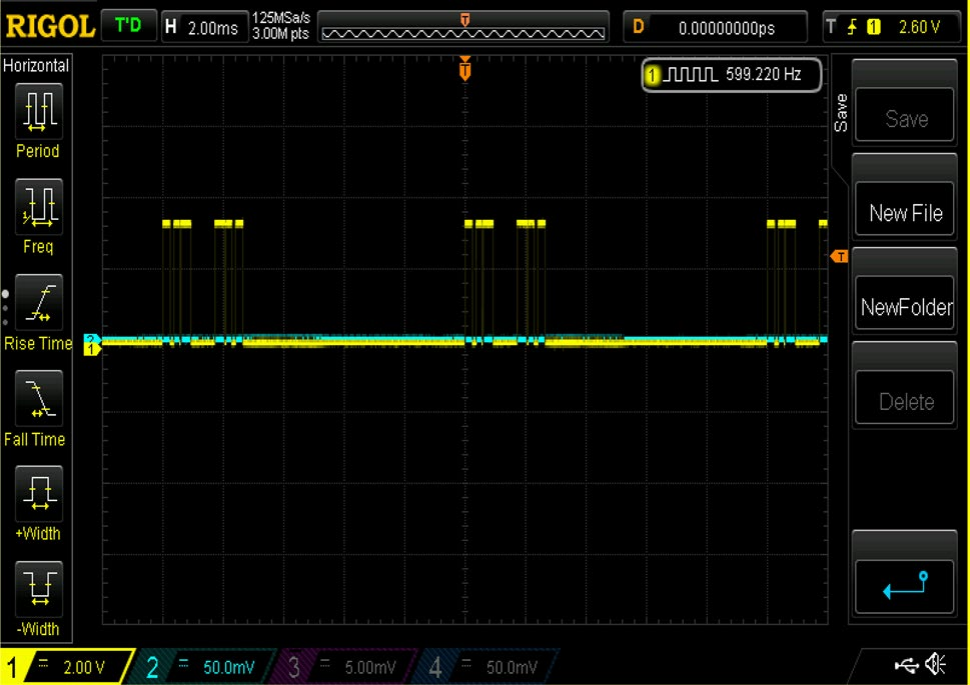
Experimental gating pulses for switch S4.

**Figure 24:**
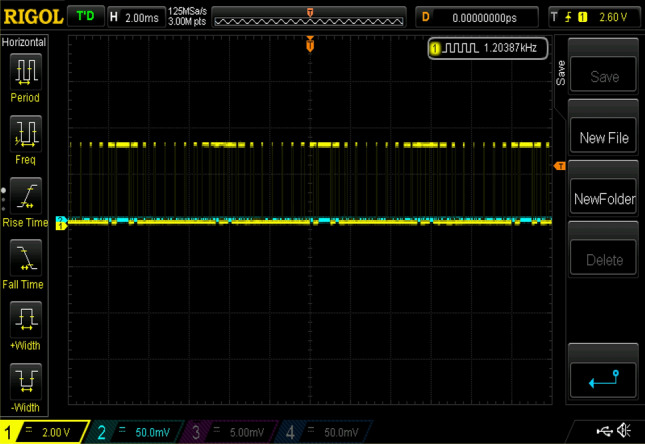
Experimental gating pulses for switch S5.

**Figure 25:**
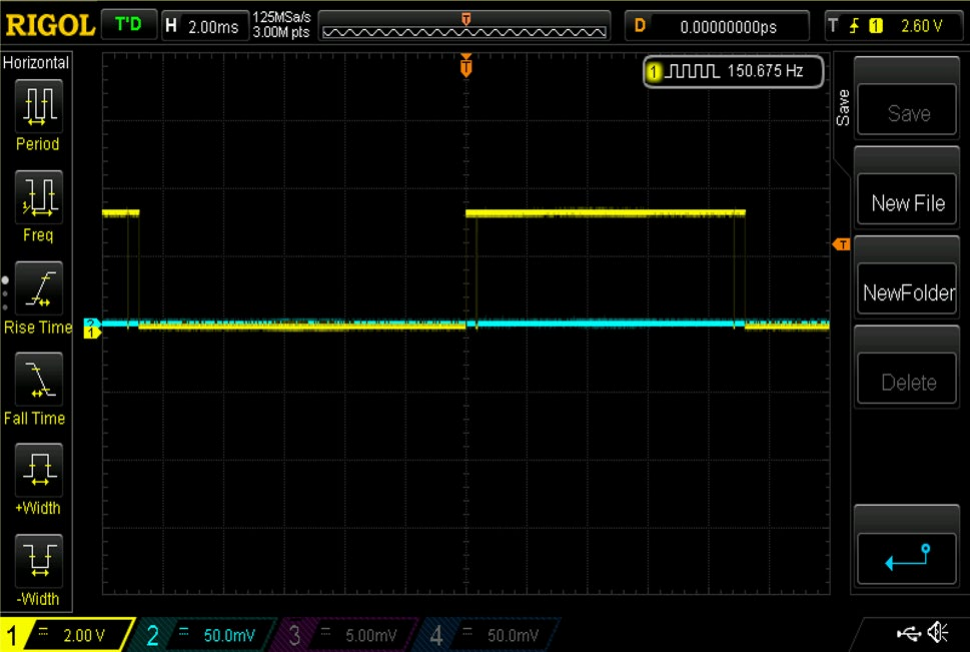
Experimental gating pulses for switch Sa1 and Sa2.

**Figure 26:**
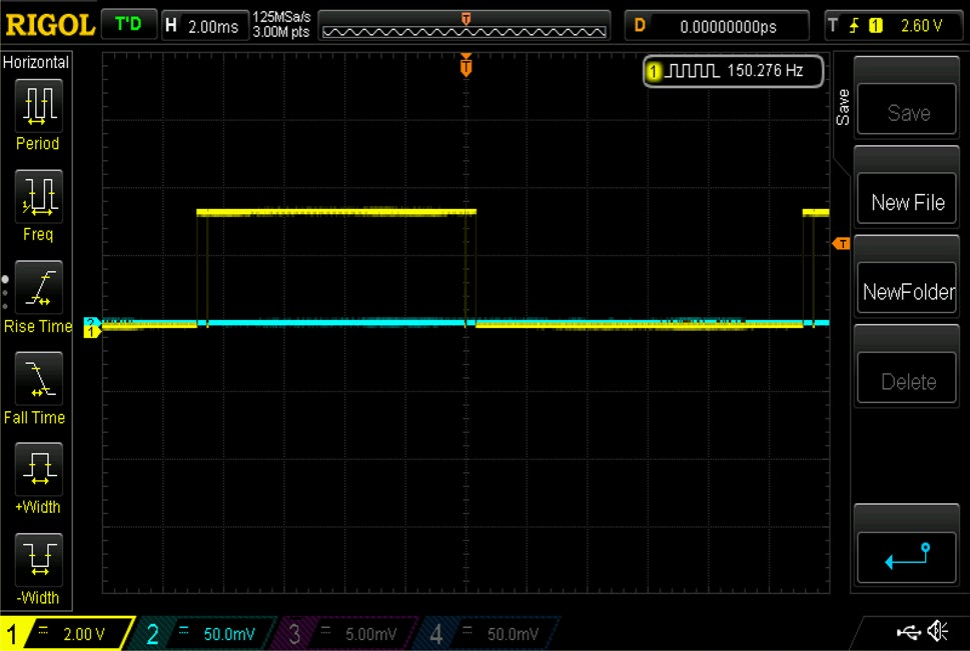
Experimental gating pulses for switch Sa3 and Sa4.

Experimental gating pulses for switch S1, switch S2, switch S3, switch S4, and switch S5 are shown in Figs. 20, 21, 22, 23 and 24 respectively. Similarly Figs. 25 and 26 shows the gating pulses for switch Sa1 and Sa2 and Sa3 and Sa4 respectively acquired from the experimental results.

## Conclusions

A novel MLI structure has been constructed with lesser power devices, and gate drivers along with total current conducting components for applications under low/medium voltages. The design operates with fewer switches in the current route by using multiple components consecutively. The suggested MLI necessitates fewer switches as well as DC sources compared to cascaded MLIs. The PWM pulse generation uses an FPGA controller that makes a flexible way extension for three-phase cascaded MLIs. The experimental investigation recommends further possibilities in developing new MLI structures for clean energy and variable speed drive applications that may utilize reduced power components.

## Data Availability

All data generated or analysed during this study are included in this article.
